# KL-6 concentration in pulmonary epithelial lining fluid is a useful prognostic indicator in patients with acute respiratory distress syndrome

**DOI:** 10.1186/1465-9921-12-32

**Published:** 2011-03-22

**Authors:** Tomohiro Kondo, Noboru Hattori, Nobuhisa Ishikawa, Hiroshi Murai, Yoshinori Haruta, Nobuyuki Hirohashi, Koichi Tanigawa, Nobuoki Kohno

**Affiliations:** 1 Department of Molecular and Internal Medicine, Graduate School of Biomedical Sciences, Hiroshima University, 1-2-3 Kasumi, Minami-ku, Hiroshima, 734-8551, Japan; 2 Department of Emergency and Critical Care Medicine, Hiroshima University Hospital, 1-2-3 Kasumi, Minami-ku, Hiroshima, 734-8551, Japan

## Abstract

**Background:**

KL-6 is a mucin-like glycoprotein expressed on the surface of alveolar type II cells. Elevated concentrations of KL-6 in serum and epithelial lining fluid (ELF) in patients with acute respiratory distress syndrome (ARDS) have been previously reported; however, kinetics and prognostic significance of KL-6 have not been extensively studied. This study was conducted to clarify these points in ARDS patients.

**Methods:**

Thirty-two patients with ARDS who received mechanical ventilation under intubation were studied for 28 days. ELF and blood were obtained from each patient at multiple time points after the diagnosis of ARDS. ELF was collected using a bronchoscopic microsampling procedure, and ELF and serum KL-6 concentrations were measured.

**Results:**

KL-6 levels in ELF on days 0 to 3 after ARDS diagnosis were significantly higher in nonsurvivors than in survivors, and thereafter, there was no difference in concentrations between the two groups. Serum KL-6 levels did not show statistically significant differences between nonsurvivors and survivors at any time point. When the highest KL-6 levels in ELF and serum sample from each patient were examined, KL-6 levels in both ELF and serum were significantly higher in nonsurvivors than in survivors. The optimal cut-off values were set at 3453 U/mL for ELF and 530 U/mL for serum by receiver operating characteristic (ROC) curve analyses. Patients with KL-6 concentrations in ELF higher than 3453 U/mL or serum concentrations higher than 530 U/mL had significantly lower survival rates up to 90 days after ARDS diagnosis.

**Conclusions:**

ELF and serum KL-6 concentrations were found to be good indicators of clinical outcome in ARDS patients. Particularly, KL-6 levels in ELF measured during the early period after the diagnosis were useful for predicting prognosis in ARDS patients.

## Background

Acute respiratory distress syndrome (ARDS) is characterized by the influx of protein-rich edema fluid into air spaces because of the increased permeability of the alveolar-capillary barrier [1,2]. The important roles of endothelial injury and increased vascular permeability in the formation of pulmonary edema have been well established in this disorder [3]. An intact alveolar epithelial barrier is necessary for preventing alveolar flooding and facilitating recovery from ARDS; therefore, the degree of alveolar epithelial injury is an important predictor of the outcomes in ARDS [4-6]. When epithelial integrity is lost and alveolar type II cells are injured, normal alveolar epithelial fluid transport and removal of alveolar edema fluid are impaired [7]. Moreover, injury to alveolar type II cells reduces the production and turnover of surfactant [8], and may also cause intrapulmonary bacterial translocation that may lead to bacteremia or sepsis [9]. If injury to the alveolar epithelium is severe, epithelial repair is impaired, which may lead to the development of fibrosis [10].

KL-6 is a high-molecular-weight glycoprotein, classified according to immunohistochemical and flow cytometry study findings as cluster 9 mucin-1 (MUC1) of lung tumor and differentiation antigens [11]. After cleavage of the S-S bond near the surface of the epithelial cell membrane, KL-6 can diffuse into pulmonary epithelial lining fluid (ELF). In the normal lung, this glycoprotein can be predominantly found on alveolar type II epithelial cells, and its expression is greatly increased in proliferating, regenerating, or injured alveolar type II cells [12-14]. Previous studies have demonstrated that serum levels of KL-6 are elevated in a variety of interstitial lung diseases that are characterized by alveolar epithelial cell damage [12,14-20]. Because serum levels of KL-6 have been shown to be correlated with indices of alveolar-capillary permeability [15], elevated levels of circulating KL-6 are believed to be associated with its increased leakage from the alveolar space into the circulation.

Previous studies examined KL-6 levels in the serum and pulmonary ELF or bronchoalveolar lavage fluid (BALF) of adult patients with ARDS or acute lung injury (ALI) [13,21-23], and found that the levels of KL-6 were significantly elevated. These studies also reported that the levels of KL-6 in these samples were significantly higher in nonsurvivors than in survivors. Their results suggest that elevated levels of KL-6 may indicate poor prognosis in ARDS patients; however, whether or not KL-6 levels in these samples can predict clinical outcomes in ARDS patients has not yet been studied in detail. Furthermore, none of these studies have reported detailed kinetics of KL-6 levels in ELF and serum in ARDS patients.

In the present study, to further evaluate the clinical significance of KL-6 in ARDS patients, concentrations of KL-6 in ELF and serum were consecutively measured in 32 patients who developed ARDS in our hospital, and the kinetics of KL-6 levels in ELF and serum during 4 weeks after the diagnosis of ARDS were determined. In addition, the associations between KL-6 levels in these samples and patient clinical outcomes were examined.

## Methods

### Study population and protocol

This clinical study was conducted at Hiroshima University Hospital between July 2007 and March 2009. The human research committee of Hiroshima University approved this study, and written informed consent was obtained from each study participant or from immediate family members. Thirty-two patients were prospectively diagnosed with ARDS according to the definition of the American-European Consensus Conference on ARDS. They were included in the study if they met consensus conference oxygenation and radiographic criteria for ARDS, and were followed until death or hospital discharge. The patients who were discharged from the hospital were considered to be survivors.

Bronchoscopic microsampling (BMS) of ELF was performed on days 0, 1, 3, 5, 7, 10, 14, 21, and 28 in each patient unless the patient had been extubated or had died. The first sample was taken on day 0, within 24 hours after the diagnosis of ARDS. In addition, blood was sampled on days 0, 1, 3, 5, 7, 10, 14, 21, and 28.

### BMS procedure

All studied patients were sedated and preoxygenated (FiO_2 _= 1.0). A flexible bronchoscope (BF-6C240; Olympus, Tokyo, Japan) was inserted into the lung through an intratracheal tube to examine the airway, and any excess sputum was suctioned. Another identical bronchoscope was then inserted and its tip was advanced into a segmental bronchus of the right middle lobe (S4 or S5), and the BMS procedure was performed as described previously [24]. The BMS probe (Olympus, Tokyo, Japan), consisted of a polyethylene outer sheath 1.7 mm in diameter and an inner fiber rod probe 1.2 mm in diameter and 30 mm in length, attached to a stainless steel guide wire 100 cm in length. Briefly, the probe was inserted into the channel and gently advanced. While the outer sheath was set at the target in the subsegmental bronchus, the inner probe was advanced slowly into the peripheral airway until it contacted the mucosal surface, and it was held in that position for 5-7 seconds, thus allowing the fiber rod to absorb approximately 20 μL of ELF. The inner probe was then withdrawn into the outer sheath, and they were removed together. The wet inner probe was cut, placed in a tube, and stored in a freezer at -80°C until analysis. The procedure was performed in triplicate from the same subsegmental bronchus.

The stored frozen probes were weighed before the ELF saline suspension was prepared. Diluted ELF sample solutions were prepared for biochemical analysis by adding the 3 frozen probes that had been sampled from the same lung subsegment to a 15 mL polyethylene tube containing 3 mL of saline, which was then vortexed for 1 minute. The solution was centrifuged for 15 minutes at 3,000 rpm, and the supernatant was collected. The probes were dried and weighed to calculate the ELF volume recovered. The dilution factor was calculated as follows: ELF volume (mL)/(3 mL + ELF volume [mL]).

In vitro experiments have confirmed that the absorption of 2-20 μL of human serum by the fiber rod probe allowed a >93% recovery of biochemical constituents. The recovery was 96.1% for albumin, 93.7% for lactate dehydrogenase (LDH), and 95.3% for KL-6.

### Measurements of KL-6

KL-6 levels in the serum and ELF samples were measured by a sandwich-type electrochemiluminescent immunoassay (ECLIA) using a Picolumi 8220 Analyzer (Sanko Junyaku, Tokyo, Japan), as previously described [25]. In brief, the sample was incubated with anti-KL-6 antibody-coated magnetic beads and the beads were then separated using a magnetic rack. Ruthenium-labeled anti-KL-6 antibody was added to the beads as a second antibody, following a PBS wash. The reaction mixture was placed into an electrode, and the photons emitted from the ruthenium were measured by a photomultiplier.

### Statistical analysis

Statistical significance was defined as *p *< 0.05. Differences in variables between survivors and nonsurvivors were compared using the nonparametric Mann-Whitney *U*-test, since the data were not normally distributed. The variables at each time point in survivors and nonsurvivors during 4 weeks after the diagnosis of ARDS were compared using both one-way analysis of variance (ANOVA) and test for linear trend with multiple comparisons. Receiver operating characteristic (ROC) curve analysis was used to assess KL-6 in ELF as a prognostic indicator in ARDS patients. Survival until 90 days after the diagnosis was evaluated by the Kaplan-Meier method. The difference in survival between two groups was analyzed by the log-rank test. All patients included into the study were followed-up until 90 days after the diagnosis of ARDS.

## Results

### Characteristics of patients

Thirty-two consecutive patients with ARDS who were treated with controlled mechanical ventilation in the intensive care unit were studied between July 2007 and March 2009. The primary disorders in these patients were pneumonia (n = 10), sepsis (n = 10), gastric aspiration (n = 5), liver failure (n = 2), alveolar hemorrhage (n = 1), interstitial pneumonia (n = 1), hypersensitivity pneumonia (n = 1), drug-induced pneumonia (n = 1), and chest trauma (n = 1). The patients with interstitial pneumonia, hypersensitivity pneumonia, and drug-induced pneumonia were confirmed to have had stable respiratory condition before the onset of ARDS and the apparent superimposition of pulmonary infection in these three patients was denied by the analysis of BALF. The mean age (± SD) was 70.1 ± 11.7 years, and 27 patients were males. The initial mean value (± SD) for PaO_2_/FIO_2 _was 108.6 ± 39.8, and the in-hospital mortality rate was 31.3%.

### KL-6 levels in ELF and serum samples of survivors and nonsurvivors

**Figure 1:**
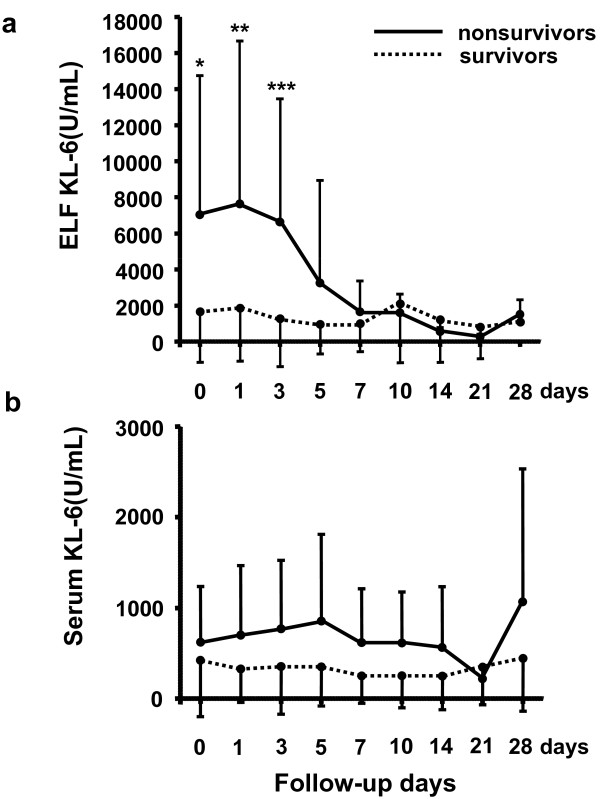
**Kinetics of KL-6 levels in ELF (a) and serum (b) in the nonsurvivors (n = 10) and survivors (n = 22)**. Data are means ± SD. *p = 0.0087, **p = 0.0421, ***p = 0.0324 by Mann-Whitney *U*-test. A significant reducing trend in ELF levels of KL-6 was observed in the nonsurvivors (*p *= 0.0318 by test for linear trend).

**Figure 2:**
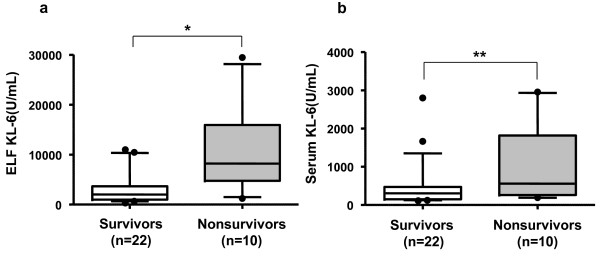
**Comparisons of the highest KL-6 levels in ELF (a) and serum (b) from the serial measurements of the nonsurvivors and survivors**. The Box-whisker plots show the 25th and 75th percentiles, the median (horizontal line within the box), and the 10th and 90th percentiles (whiskers). *p = 0.0025, **p = 0.0401 by Mann-Whitney *U*- test.

The kinetics of KL-6 levels in ELF and serum samples were first compared between the survivors and nonsurvivors. The KL-6 levels in ELF were significantly higher in nonsurvivors than in the survivors on days 0 (*p *= 0.0087), 1 (*p *= 0.0421), and 3 (*p *= 0.0324) (Figure 1a). The variables at each time point were compared in the survivors and nonsurvivors using one-way ANOVA and no statistical differences were found in each comparison. However, only in the nonsurvivors, a reducing trend in ELF levels of KL-6 as time passed after the diagnosis of ARDS was observed (test for linear trend, *p *= 0.0318). There were no significant differences seen in serum KL-6 levels between survivors and nonsurvivors at any time point throughout the clinical courses of the patients (Figure 1b). To obtain more information on the clinical significance of KL-6 in ARDS, we selected the highest ELF and serum KL-6 concentrations among the series of measurements in each patient and compared the results between survivors and nonsurvivors. The highest concentrations of KL-6 in ELF were observed on days 2.7 ± 3.3 in the nonsurvivors; whereas the peak levels in the survivors occurred on days 3.6 ± 4.4. The mean highest concentrations of KL-6 in ELF were 10733.6 ± 7793.1 U/mL in the nonsurvivors and 3282.3 ± 3474.1 U/mL in the survivors. The highest concentrations of KL-6 in serum were observed on days 5.8 ± 8.4 in the nonsurvivors; whereas the peak levels in the survivors occurred on days 2.6 ± 4.5. The mean highest concentrations of KL-6 in serum were 1060.8 ± 989.8 U/mL in the nonsurvivors and 466.8 ± 602.1 U/mL in the survivors. The highest KL-6 levels in ELF and serum were significantly higher in the nonsurvivors than in the survivors (*p *= 0.0025, Figure 2a; and *p *= 0.0401, Figure 2b; respectively). In addition to the comparisons of the KL-6 levels between the survivors and nonsurvivors, the highest KL-6 levels in ELF and serum among the series of measurements were compared between the patients with primary (n = 20) and secondary (n = 12) ARDS or between the patients with (n = 3) and without (n = 29) preexisting interstitial lung disease (ILD). In each comparison, we found no significant difference between the two groups of the patients (data not shown).

### Prognostic values of KL-6 levels in pulmonary ELF and serum obtained from ARDS patients

**Figure 3:**
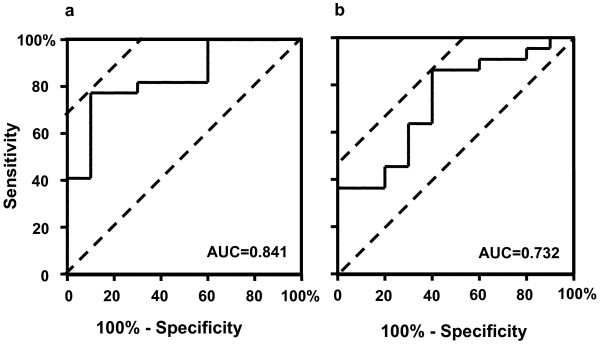
**ROC curve analyses to determine the optimal cutoff values of KL-6 concentrations in ELF (a) and serum (b) for predicting survival in ARDS patients**. The highest KL-6 levels in ELF and serum from each patient were used for the analysis. The vertical axis represents the number of true-positive responses (sensitivity), and the horizontal axis represents the number of false-positive responses (100%-specificity). The area under the curve (AUC) represents the fraction of nonsurviving ARDS patients who would have a positive test (high KL-6 concentration in ELF or serum). The optimal value of KL-6 in ELF was 3453 U/mL, with a sensitivity, specificity, and likelihood ratio of 77.27%, 90.0%, and 7.73, respectively. The optimal value of KL-6 in serum was 530 U/mL, with a sensitivity, specificity, and likelihood ratio of 86.36%, 60.0%, and 2.16, respectively.

**Figure 4:**
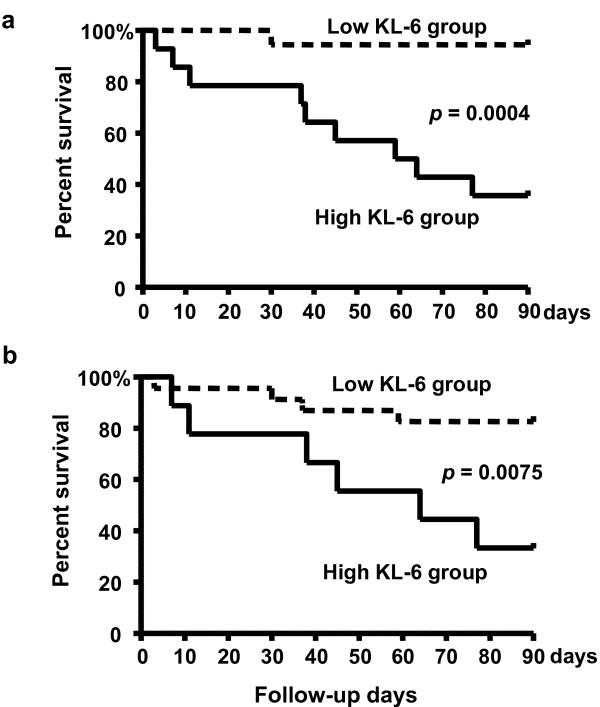
**Overall survival of ARDS patients in relation to KL-6 concentrations in ELF (a) and in serum (b)**. The survival rate of patients with a high KL-6 levels in ELF and serum was significantly lower than that of patients with a low KL-6 levels (ELF: *p *= 0.0004, serum: *p *= 0,0075 by log-rank test).

To obtain optimal cut-off values for KL-6 in ELF and serum for prognostic assessment in ARDS patients, receiver operating characteristic (ROC) curve analyses were performed using the highest concentrations of KL-6 measured in the serial ELF (Figure 3a) and serum (Figure 3b) samples. For predicting the risk of mortality, the optimal cut-off value for KL-6 in ELF was 3453 U/mL, with a sensitivity, specificity, and likelihood ratio of 77.27%, 90.0%, and 7.73, respectively. Nine out of 14 patients with ELF KL-6 levels > 3453 U/mL died; whereas only 1 death was observed in the 18 patients with ELF KL-6 levels < 3453 U/mL died (*p *= 0.0006). The optimal cut-off value of KL-6 in serum was found to be 530 U/mL, with a sensitivity, specificity, and likelihood ratio of 86.36%, 60.0%, and 2.16, respectively. Whereas 6 out of 9 patients with serum KL-6 levels > 530 U/mL died, only 4 out of 23 patients with serum KL-6 levels < 530 U/mL died (*p *= 0.0126). Based on these cut-off values, overall survivals up to 90 days after the diagnosis of the ARDS were determined using the Kaplan-Meier method. The survival of patients with concentrations of KL-6 in ELF higher than 3453 U/mL was significantly poorer than the survival of patients with lower KL-6 concentrations (*p *= 0.0004, Figure 4a). Similarly, the survival of patients with higher serum KL-6 levels (> 530 U/mL) was significantly poorer than the survival of patients with lower serum KL-6 levels (*p *= 0,0075, Figure 4b).

## Discussion

In this study, we measured KL-6 concentrations in pulmonary ELF samples and serum samples obtained at multiple time points from ARDS patients. When the kinetics of KL-6 levels in ELF and serum were compared between the survivors and nonsurvivors, only the levels of KL-6 in ELF on days 0 to 3 after the diagnosis of ARDS were significantly higher in the nonsurvivors than in the survivors. There were no differences between survivors and nonsurvivors in KL-6 concentrations in ELF samples at other time points, and there were no significant differences in serum KL-6 levels between the survivors and nonsurvivors at any time point. However, when the highest serum KL-6 levels from the serial samples from each patient were compared between the survivors and nonsurvivors, statistically significant higher serum KL-6 levels were seen in the nonsurvivors. In addition, KL-6 levels in ELF higher than 3453 U/mL and KL-6 levels in serum higher than 530 U/mL were shown to be significant prognostic factors for predicting poor overall survival up to 90 days after the diagnosis of ARDS.

The most important finding in the present study was that the marked elevation of ELF KL-6 within 3 days after the diagnosis appeared to correlate with poor prognosis in ARDS patients. This observation was supported by the following study results: KL-6 levels in ELF were significantly elevated in the nonsurvivors on days 0 to 3 after the diagnosis of ARDS compared to the survivors, and the patients with KL-6 levels in ELF higher than 3453 U/mL had significantly poorer prognosis than those with lower KL-6 levels in ELF. Lung compartment KL-6 is believed to be produced and released by proliferating alveolar type II cells following injury to alveolar type I cells [21], and therefore its level must reflect the severity of alveolar epithelial injury. The degree of alveolar epithelial injury is believed to be an important predictor of outcomes in patients with ARDS [2,26]. Based on these concepts, a very high KL-6 level in ELF can be regarded as an indicator of very severe alveolar epithelial damage, and a predictor of poor prognosis in ARDS. In turn, our data suggest that measurement of KL-6 levels in ELF, particularly during the early period after ARDS diagnosis, is useful for assessing the degree of alveolar epithelial damage and predicting overall clinical outcome.

Another interesting finding was that in the nonsurvivors, the significantly elevated levels of KL-6 in ELF were only observed on days 0 to 3 after ARDS diagnosis, and thereafter, the levels of KL-6 in ELF were similar to the levels in the survivors. In fact, the highest concentrations of KL-6 in ELF were observed on days 2.7 ± 3.3 in the nonsurvivors; whereas in the survivors, they occurred on days 3.6 ± 4.4. Therefore, we can suggest that at least one BMS procedure within 3 days after the diagnosis of ARDS is sufficient to predict the clinical outcome and the KL-6 levels in ELF obtained from 4 days after the diagnosis may have less impact on the prediction of prognosis. Unfortunately, we do not have convincing data to explain why levels of KL-6 in ELF in the nonsurvivors dropped to the same levels as those in the survivors. It has been suggested that alveolar type II cells can proliferate when alveolar epithelial cell damage is mild or moderate, but when the damage is very severe, even type II cells cannot survive and are replaced by the epithelial cells of bronchial origin [27,28]. Furthermore, if the alveolar epithelial injury is too severe for recovery, insufficient or disorganized epithelial repair occurs, resulting in the development of fibrosis [2]. Based on these concepts, we can speculate that in the nonsurvivors, the alveolar type II cells could initially proliferate during the early stages of ARDS, leading to elevated KL-6 pulmonary ELF concentrations; however, after development of severe alveolar epithelial damage, the type II cells died or disorganized epithelial repair occurred, leading to decrease in level of KL-6 in ELF.

In contrast to the results of previous reports [13,22,23], there were no statistically significant differences in serum KL-6 levels between the nonsurvivors and survivors observed at any time points among the serial measurements. Serum KL-6 levels at each time point tended to be higher in the nonsurvivors than in the survivors; therefore we believe that if our study would be larger, statistically significant differences could have been seen. Indeed, when the highest serum level of KL-6 from the serial measurements in each patient was used for comparisons, it was significantly higher in the nonsurvivors than in the survivors. In addition, the patients with the highest serum KL-6 levels that were higher than 530 U/mL were found to have poorer prognosis than the other patients. In children with ARDS, circulating levels of KL-6 were also reported to be higher in the nonsurvivors than the survivors [29]. These data suggest that serum KL-6 concentrations also reflect the degree of alveolar epithelial injury and may be useful for predicting clinical outcomes in patients with ARDS. However, we believe that the concentration of KL-6 in ELF is a more sensitive indicator of alveolar epithelial injury, and is thus a more useful predictor of clinical outcome than the serum KL-6 level, because it provides more immediate information on events taking place in the lung.

Because KL-6 is mainly expressed in alveolar type II epithelial cells and a sensitive biomarker to detect the presence of ILD, we questioned whether there was a difference in KL-6 levels in ELF and serum between the patients with primary and secondary ARDS or between the patients with and without preexisting ILD. Interestingly, we found no significant difference in each comparison. These data suggest that KL-6 levels in ELF and serum were not affected by the cause of ARDS. In addition, the presence of preexisting ILD seemed not to influence the KL-6 levels in ELF and serum after developing ADRS. However, we believe that the number of cases with preexisting ILD was too small (only three) to reach the latter conclusion and, therefore, further study on this issue is necessary.

Although promising results were obtained, we are aware that this study has some limitations. The number of patients included in the study was not sufficient to confirm previous observations that circulating KL-6 levels were significantly higher in nonsurvivors than survivors, particularly during the early period after the onset of ARDS [13,22,23]. The BMS procedure has an intrinsic limitation, in that exploratory sampling in the lung is limited. Additional study measuring KL-6 in ELF from different sampling sites in the lungs of each ARDS patient is necessary.

## Conclusion

Concentrations of KL-6 in pulmonary ELF early after ARDS diagnosis were found to be significantly higher in nonsurviving patients than in surviving patients. Furthermore, ARDS patients with higher KL-6 levels in ELF or serum had significantly poorer prognosis than those with lower KL-6 levels. The levels of KL-6 in ELF and serum may reflect the degree of alveolar epithelial injury, and may therefore be valuable indicators of outcome in ARDS. Particularly, the concentration of KL-6 in ELF measured during the early period after the diagnosis appears to be a useful marker for predicting prognosis in ARDS patients.

## List of abbreviations

ALI: acute lung injury; ANOVA: analysis of variance; ARDS: acute respiratory distress syndrome; AUC: area under the curve; BALF: bronchoalveolar lavage fluid; BMS: bronchoscopic microsampling; ELF: epithelial lining fluid; ILD: interstitial lung disease; ROC: receiver operating characteristic

## Competing interests

The authors declare that they have no competing interests.

## Authors' contributions

TK designed the study, performed the data analysis and interpretation, and wrote the manuscript. NH and NI designed the study, interpreted the data, and edited the manuscript. HM, YH, NH, KT, and NK interpreted the data and helped to draft the manuscript. All authors read and approved the final manuscript.
